# Chemistry glows green with photoredox catalysis

**DOI:** 10.1038/s41467-019-13887-8

**Published:** 2020-02-06

**Authors:** Giacomo E. M. Crisenza, Paolo Melchiorre

**Affiliations:** 1 ICIQ, Institute of Chemical Research of Catalonia - the Barcelona Institute of Science and Technology, Av. Països Catalans 16, 43007 Tarragona, Spain; 2 ICREA, Catalan Institution for Research and Advanced Studies, Passeig Lluís Companys 23, 08010 Barcelona, Spain

**Keywords:** Synthetic chemistry methodology, Photocatalysis, Process chemistry

## Abstract

Can organic chemistry mimic nature in efficiency and sustainability? Not yet, but recent developments in photoredox catalysis animated the synthetic chemistry field, providing greener opportunities for industry and academia.

## Light on sustainability

Nature is the main inspiration for scientists when it comes to sustainability. In biology, plants use photosynthesis to convert raw materials (CO_2_ and water) into chemical energy (carbohydrates), exploiting the energy of solar photons. Photosynthesis is the quintessence of sustainable chemical reactivity, and the pinnacle that Green Chemistry aims to reach. Perhaps, one step forward in this direction has been made by recent progress in photochemical methods, particularly photoredox catalysis^1^. Working as aspiring leaves, these strategies generate reactive radicals by using the ability of coloured catalysts (transition metal complexes or organic dyes) to absorb visible light radiation and activate stable low-energy organic molecules through single-electron processes (oxidation or reduction). These open-shell intermediates have been used to design a variety of reactions, which are unattainable with classical ionic chemistry triggered by thermal activation. Importantly, photoredox catalysis has revitalised other areas of synthesis, such as radical chemistry and photochemistry, providing opportunities for reaction invention and improvement. This chemistry has been used to tackle longstanding challenges in medicinal chemistry^2^, natural product synthesis^3^, and more broadly, across the spectrum of organic chemistry and catalysis^1,4^. Along with its synthetic advantages, photoredox catalysis has clear benefits for sustainability, fulfilling several principles of Green Chemistry^5^. Light radiation is its primary energy source. Light is free, non-hazardous, and environmentally friendly (energy efficiency). Photons provide enough energy to achieve the desired reactivity, without the high temperatures or harsh conditions often required by thermal activation. The light-absorbing species (photocatalysts) can be used in low catalytic amounts (use of catalytic reagents). By reaching an electronically excited state, they trigger single-electron transfer (SET) events to or from inactive/stable substrates. This generates highly reactive species in a mild and controlled manner. This has two positive impacts on sustainability. First, one can use less reactive low-energy reagents, allowing less hazardous and safer synthetic routes and easier disposal of less toxic or polluting by-products. Second, photoredox catalysis can activate generally poor reactive moieties within molecules (e.g. C–H bonds), while showing heightened functional group tolerance. This makes photoredox catalysis invaluable for designing shorter synthetic routes with enhanced atom economy, using renewable feedstock materials.

## Revived opportunities for drug discovery

**Fig. 1:**
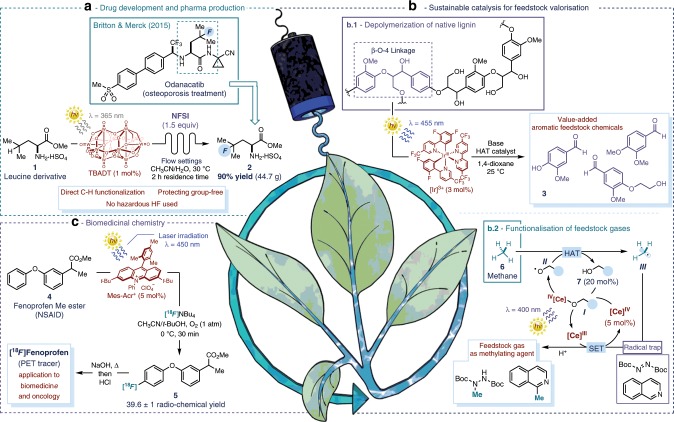
Sustainable photoredox strategies. **a** Drug development (synthesis of key drug intermediates), **b** sustainable catalysis for the valorisation of renewable feedstock (lignin biomass depolymerisation and direct functionalisation of natural gases), **c** biomedicinal chemistry (provision of radiolabelled tracers). TBADT: tetra-n-butylammonium decatungstenate; NFSI: N-fluorobenzenesulfonimide; HAT: hydrogen atom transfer; NSAID: nonsteroidal anti-inflammatory drug; PET: positron emission tomography; SET: single electron transfer.

All these features have attracted the attention of the chemical industry, which recognised the potential of photoredox strategies to achieve efficient and sustainable catalysis. For example, the pharmaceutical industry has used photoredox catalysis in several synthetic transformations that are crucial for drug discovery and development^6^. One example is protocols for the direct and selective functionalisation of drug-like scaffolds (e.g. alkylation, amination, halogenation, perfluoroalkylation). Photoredox methods have been used to install such small functionalities to directly influence the ADME-tox (absorption, distribution, metabolism, excretion, and toxicology) features of a lead candidate^7^, using non-toxic and readily available materials^8^. Within this context, the Britton group and Merck collaborated to develop a photocatalytic C-H fluorination method for the preparative provision of γ-fluoroleucine derivative **2**, a key intermediate in the synthesis of Odanacatib, a promising lead structure for osteoporosis treatment (Fig. 1a)^9^. Previous routes for **2** involved multistep synthesis (at least 2–3 steps) and hazardous reagents (e.g. hydrofluoric acid). Here, **2** was obtained directly from the unprotected amino acid derivative **1**, using a more user-friendly fluorine source (*N*-fluorobenzenesulfonimide, NFSI) and with high productivity, thanks to the use of flow settings (90% yield, 45 g after 2 h of residence time). The desired reactivity depends on irradiating, under ultraviolet light, a tungsten-based photocatalyst (tetra-*n*-butylammonium decatungstenate, TBADT), which can perform the selective hydrogen-atom abstraction at the *iso*-propyl moiety within **1**.

Drug development requires step-economical routes to construct larger libraries of lead analogues. Appealing transformation are those that enables the direct and rapid derivatisation of advanced drug candidate intermediates. Such strategies, referred to as late-stage functionalisation, could be successfully implemented by applying photoredox procedures, due to their selectivity and extraordinary functional group tolerance^10^. Drug discovery has also benefited from recent developments in metallaphotoredox catalysis (i.e. the combination of transition metal and photoredox catalysis)^11^, which has expanded the synthetic potential of cross-coupling reactions. This strategy allows unprecedented disconnections and the use of novel coupling partners, creating opportunities to improve the sustainability of carbon–carbon bond-forming coupling processes. Relevant benefits in terms of Green Chemistry are the circumvention of superstoichiometric amounts of reductants in reductive cross-coupling methods, the development of highly available and easily disposable functionalities (e.g. alcohols, carboxylic acid derivatives, and native C–H bonds) as precursors in C(sp^3^)–C(sp^2^) and C(sp^3^)–C(sp^3^) couplings, in place of less stable halogenated compounds, and the use of earth-abundant transition metal sources. For example, nickel-catalysed^11^ and copper-catalysed^12^ photochemical cross-coupling methodologies have been successfully implemented, although a natural direction for this research area would be to achieve more sustainable iron and cobalt catalysis.

## Sustainable catalysis for valorisation of renewable sources

The above examples highlight the benefits offered by photoredox catalysis in terms of mass-based metrics (improved E factor and atom economy for shorter synthetic routes). Photoredox chemistry also positively influences industrial production in terms of improved energy efficiency (milder conditions), environmental impact (reduced waste), and use of renewable resources^5^. This aspect is highlighted by the implementation of photochemical methods for the revalorisation of bulk biomass and feedstock materials. For example, Knowles and co-workers have recently obtained value-added aromatic feedstock chemicals, such as **3** (Fig. 1b, top), by exposing native lignin samples (derived from the sawdust of different plants) to irradiation with blue-light-emitting diodes (LEDs) in the presence of catalytic amounts of an iridium-based photocatalyst, a base, and a hydrogen atom transfer (HAT) thiol catalyst^13^. Along the same line, Zuo and co-workers developed a catalytic system, which combined simple organic alcohols **7** (e.g. trichloroethanol or methanol) and inexpensive cerium sources, enabling the direct functionalisation of hydrocarbon feedstocks, such as methane **6**^14^. As depicted in Fig. 1b (bottom), the transient Ce(IV)-alkoxide intermediate **I** is formed upon the interaction of **7** and the cerium precatalyst. Under violet light irradiation, **I** undergoes homolysis, generating alkoxy radical **II**. Then, **II** triggers the HAT event, generating open-shell radical **III** from **6**. This method converts widely available natural gases into effective alkylating agents for nitrogen compounds and heterocycles. Both strategies depicted in Fig. 1b convert feedstock mass into useful synthetic building blocks, using only simple catalytic agents and light radiation, demonstrating the potential of photoredox methods for circular chemistry^15^.

## Looking at a brighter future

Despite these achievements, several challenges must be addressed to secure further progress in photochemistry and to facilitate the use of photoredox strategies in industrial settings. In particular, photoredox protocols have generally long reaction times (in the range of hours instead of minutes) and limited scalability (due to the need for an efficiently illuminated surface-to-volume ratio), thwarting their application in high-volume industrial production. Flow techniques have provided promising solutions^16^, although more efficient process-scale photoreactors are still required. A second issue is the extensive use of precious metal complexes as photocatalysts. They are available in only limited amounts within the Earth’s crust. Some engineered organic dyes have achieved comparable efficiency to iridium- or ruthenium-based catalysts^17^, intensifying their use in photoredox protocols^8,18^. For example, the Nicewicz group designed an acridinium-based organic photocatalyst (**Mes-Acr**^**+**^, in Fig. 1c) able to oxidise electron-rich aromatics under irradiation from a blue-emitting laser, thus making them prone to nucleophilic attack by a mild fluorine source, such as tetra-*n*-butylammonium fluoride (FNBu_4_)^19^. In a collaboration with the Li group, this metal-free strategy was used for the selective ^18^F-fluorination of nonsteroidal anti-inflammatory drugs (NSAID) such as the Fenopren derivative **4**. Using a radio-labelled fluoride source, ^18^F-compound **5** was obtained in consistent radiochemical yield. The reaction time of 30 min is within the timeframe of the isotopic decay of ^18^F. As such, **5** can be converted by simple hydrolysis into [^18^F]Fenopren and used as a tracer for positron emission tomography (PET), with potential applications in biomedicine and oncology. Nevertheless, the main barrier to the widespread use of organic photocatalysts is their accessibility, which often requires long and elaborate syntheses. The recent development of methods exploiting the photochemical activity of readily available molecules, such as solvents or simple additives, is providing viable solutions in this regard^20^.

In conclusion, photoredox catalysis can provide efficient greener opportunities for industrial and academic research. Ideally, for greater energy efficiency and atom economy, photoredox catalysis would use both solar^21^ and direct photochemistry^22^, which can provide for faster processes while avoiding the need for artificial light sources and exogenous catalytic entities. We have not yet fully emulated the leaves of a plant, the most primitive yet most efficient photoreactors, but recent developments in photochemistry suggest that the future will be bright and green.

## References

[1] Shaw MH, Twilton J, MacMIllan DWC (2016). Photoredox catalysis in organic chemistry. J. Org. Chem..

[2] Douglas JJ, Sevrin MJ, Stephenson CRJ (2016). Visible light photocatalysis: applications and new disconnections in the synthesis of pharmaceutical agents. Org. Process Res. Dev..

[3] Nicholls TP, Leonori D, Bissember AC (2016). Applications of visible light photoredox catalysis to the synthesis of natural products and related compounds. Nat. Prod. Rep..

[4] McAtee RC, McClain EJ, Stephenson CRJ (2019). Illuminating photoredox catalysis. Trends Chem..

[5] Sheldon RA (2018). Metrics of green chemistry and sustainability: past, present, and future. ACS Sustain. Chem. Eng..

[6] Blakemore DC (2018). Organic synthesis provides opportunities to transform drug discovery. Nat. Chem..

[7] Pliska, V., Testa, B. & Waterbeemd, H. (eds). *Lipophilicity in Drug Action and Toxicology* (Wiley-VCH, Weinheim, 2008).

[8] Bogdos MK, Pinard E, Murphy JA (2018). Applications of organocatalysed visible-light photoredox reactions for medicinal chemistry. Beilstein J. Org. Chem..

[9] Halperin SD (2015). Development of a direct photocatalytic C−H fluorination for the preparative synthesis of odanacatib. Org. Lett..

[10] DiRocco DA (2014). Late-stage functionalization of biologically active heterocycles through photoredox catalysis. Angew. Chem. Int. Ed..

[11] Milligan JA, Phelan JP, Badir SO, Molander GA (2019). Alkyl carbon–carbon bond formation by nickel/photoredox cross-coupling. Angew. Chem. Int. Ed..

[12] Le C, Chen TQ, Liang T, Zhang P, MacMillan DWC (2018). A radical approach to the copper oxidative addition problem: trifluoromethylation of bromoarenes. Science.

[13] Nguyen, S. T., Murray, P. R. D. & Knowles, R. R. Light-driven depolymerization of native lignin enabled by proton-coupled electron transfer. Preprint at 10.26434/chemrxiv.9702377.v1 (2019).

[14] Hu A, Guo J-J, Pan H, Zuo Z (2018). Selective functionalization of methane, ethane, and higher alkanes by cerium photocatalysis. Science.

[15] Keijer T, Bakker V, Slootweg JC (2019). Circular chemistry to enable a circular economy. Nat. Chem..

[16] Cambié D, Bottecchia C, Straathof NJW, Hessel V, Noël T (2016). Applications of continuous-flow photochemistry in organic synthesis, material science, and water treatment. Chem. Rev..

[17] Joshi-Pangu A (2016). Acridinium-based photocatalysts: a sustainable option in photoredox catalysis. J. Org. Chem..

[18] Romero NA, Nicewicz DA (2016). Organic photoredox catalysis. Chem. Rev..

[19] Chen W (2019). Direct arene C–H fluorination with^18^F^−^ via organic photoredox catalysis. Science.

[20] Liu W, Li J, Querard P, Li C-J, Transition-metal-free C-C (2019). C-O, and C-N cross-couplings enabled by light. J. Am. Chem. Soc..

[21] Oelgemöller M (2016). Solar photochemical synthesis: from the beginnings of organic photochemistry to the solar manufacturing of commodity chemicals. Chem. Rev..

[22] Silvi M, Melchiorre P (2018). Enhancing the potential of enantioselective organocatalysis with light. Nature.

